# Unaccounted uncertainty from qPCR efficiency estimates entails uncontrolled false positive rates

**DOI:** 10.1186/s12859-016-0997-6

**Published:** 2016-04-11

**Authors:** Anders E. Bilgrau, Steffen Falgreen, Anders Petersen, Malene K. Kjeldsen, Julie S. Bødker, Hans E. Johnsen, Karen Dybkær, Martin Bøgsted

**Affiliations:** Department of Haematology, Aalborg University Hospital, Sdr. Skovvej 15, Aalborg, 9000 Denmark; Department of Mathematical Sciences, Aalborg University, Fredrik Bajers Vej 7G, Aalborg Ø, 9220 Denmark; Department of Clinical Medicine, Aalborg University Hospital, Sdr. Skovvej 15, Aalborg, 9000 Denmark

**Keywords:** qPCR, Amplification efficiency, Delta-delta Cq, *ΔΔC*_*q*_, Error propagation, Efficiency adjusted

## Abstract

**Background:**

Accurate adjustment for the amplification efficiency (AE) is an important part of real-time quantitative polymerase chain reaction (qPCR) experiments. The most commonly used correction strategy is to estimate the AE by dilution experiments and use this as a plug-in when efficiency correcting the *ΔΔC*_*q*_. Currently, it is recommended to determine the AE with high precision as this plug-in approach does not account for the AE uncertainty, implicitly assuming an infinitely precise AE estimate. Determining the AE with such precision, however, requires tedious laboratory work and vast amounts of biological material. Violation of the assumption leads to overly optimistic standard errors of the *ΔΔC*_*q*_, confidence intervals, and *p*-values which ultimately increase the type I error rate beyond the expected significance level. As qPCR is often used for validation it should be a high priority to account for the uncertainty of the AE estimate and thereby properly bounding the type I error rate and achieve the desired significance level.

**Results:**

We suggest and benchmark different methods to obtain the standard error of the efficiency adjusted *ΔΔC*_*q*_ using the statistical delta method, Monte Carlo integration, or bootstrapping. Our suggested methods are founded in a linear mixed effects model (LMM) framework, but the problem and ideas apply in all qPCR experiments. The methods and impact of the AE uncertainty are illustrated in three qPCR applications and a simulation study. In addition, we validate findings suggesting that *MGST1* is differentially expressed between high and low abundance culture initiating cells in multiple myeloma and that microRNA-127 is differentially expressed between testicular and nodal lymphomas.

**Conclusions:**

We conclude, that the commonly used efficiency corrected quantities disregard the uncertainty of the AE, which can drastically impact the standard error and lead to increased false positive rates. Our suggestions show that it is possible to easily perform statistical inference of *ΔΔC*_*q*_, whilst properly accounting for the AE uncertainty and better controlling the false positive rate.

## Background

Despite being an aging technique, real-time quantitative polymerase chain reaction (qPCR)—arguably one of the most significant biotech discoveries of all time—is still heavily used in molecular biology [1]. qPCR is an extremely sensitive and cost effective technique to amplify and quantitate the abundance of DNA and RNA using a Taq polymerase that for RNA analysis are preceded by a reverse transcriptase conversion into template DNA. In life sciences, qPCR is typically applied to quantify candidate gene transcripts that are biomarkers of diagnostic, prognostic, and even predictive value in e.g. infectious diseases and cancer. In the slip stream of high-volume omics-data, another very important application of qPCR has arisen. Here, qPCR is the gold standard validation tool for the most promising gene transcripts generated by high-throughput screening studies such as microarrays or sequencing. For validation experiments in particular the ability to control the type I error rate is very important. Unfortunately, important statistical details are often omitted resulting in a failure to obtain the desired type I error probability. Validation without such an ability cannot be considered very meaningful and therefore conservative approaches should be taken.

The so-called *ΔΔC*_*q*_ quantity is the normalized relative expression of a target gene of interest between treated (case) and untreated samples (control) accounting for undesired variations using one or more endogenous reference genes (also called housekeeping gene) assumed to be approximately unchanged due to the treatment. The *ΔΔC*_*q*_-value is usually based on the assumption of perfect AEs for both the target and reference gene. However, the target and reference genes might be subject to different AE which yield biased *ΔΔC*_*q*_-values. In turn, the *ΔΔC*_*q*_ has been modified to AE corrected versions [2–4].

Despite the tremendous success of qPCR, ‘statistical inference considerations are still not accorded high enough priority’ [5, 6]. We find this particular true for the estimation of the AE. Although efficiency calibration has been extensively treated by [2] or in the more generalized model by [7], there seems to be a lack of systematic studies of the unavoidable influence of the uncertainty of the AE estimate on the conclusions of qPCR experiments based on formal statistical inference. The current AE adjusted *ΔΔC*_*q*_ methods do not account for the *uncertainty of the estimated AE* and thus effectively assumes the AE to be estimated with infinite precision. This assumption entails a systematic underestimation of the standard error of *ΔΔC*_*q*_ leading to too narrow confidence intervals, decreased *p*-values, and thereby increased type I error rates. If the AE is poorly determined, this underestimation can drastically increase the standard error of *ΔΔC*_*q*_ and similar quantities.

Recently, some effort has been devoted to studying error propagation in qPCR [8–11]. Nordgaard et al. [8] studied error propagation primarily on the *C*_*q*_-values including the effect of the AE uncertainty. This study was, however, statistically informal and made no attempt to quantify the effect on the *ΔΔC*_*q*_ and inference hereof. Furthermore, they [8] considered AE estimation from the amplification curve (thus for each well) and not from separate dilution experiments. Tellinghuisen and Speiss [9–11] stressed and discussed the importance and negative impact of improper error handling, including AE estimation, although again with emphasis on determining *C*_*q*_-values and the florescence level at the hypothetical cycle zero using different methods. In this paper, we explicitly discuss only the AE estimation from dilution curves, which assumes a constant AE across certain genes. While this assumption has been contested and alternatives by branching processes suggested [12–15], the problem still exist as AE estimates from individual amplification curves also have an associated error which affect all ‘down-steam’ estimated quantities and inference. The numerous estimated well-specific AEs arguably amplify the problem as even more errors—one for each well—is propagated further on.

The work by Svec et al. [16] also recently assessed the impact of AE uncertainty as a function of the number of technical replicates at each concentration and the qPCR instrument. They conclude that a minimum of 3–4 replicates at each concentration are needed and that a significant inter qPCR instrument effect is present. However, they do not gauge the effect of the number of concentrations used—an important variable as additional technical replicates rarely contribute with much information to determine the AE. Nonetheless, Svec et al. [16] also do not address the impact of AE uncertainty on formal statistical inference on the *ΔΔC*_*q*_, as this paper intends.

### Aims

Primarily, we aim to highlight the common problem of disregarding the uncertainty of the AE estimate in statistical inference of the *ΔΔC*_*q*_-value in qPCR experiments. And we propose and benchmark different off-the-shelf and novel solutions to this problem.

To this end, we employ a statistical model which allows such formal inference. This covers statistical model formulation, confidence intervals, hypothesis testing, and power calculation, with special emphasis on false positive rates. Simultaneous estimation of the uncertainty of the AE estimate and mean *C*_*q*_-values by linear mixed effects models (LMM), which allows a more appropriate handling of the technical and sample errors, is described. We investigate the use of the statistical delta method, Monte Carlo integration, or bootstrapping to correctly perform inference on the value of *ΔΔC*_*q*_.

Note two important observations: First, the problem exists for *all* statistical models and methods which incorrectly disregard the uncertainty of the AE estimate and is not limited to LMMs. Secondly, the problem exists not only for *ΔΔC*_*q*_-values, but also all similar quantities, e.g. *ΔC*_*q*_ and *C*_*q*_, and the statistical inferences based on these.

The idea of using LMMs for qPCR experiments is not new [17–21]. For example, [17] and [18] have used mixed effects modeling to identify candidate normalizing genes. The work by [19] applied the related generalized estimation equations to handle intra and inter group variation. However, the usage of LMMs combined with the statistical delta method, Monte Carlo integration, or bootstrapping to handle uncertainty stemming from the efficiency estimation seems to be novel and provides a general statistical framework for qPCR experiments and may be considered an extension of the strategy by [7]. Others use the mixed models primarily for the *C*_*q*_-value estimation [20, 21].

We demonstrate that considering the uncertainty of the AE is, unsurprisingly, highly important when the AE is determined with inadequate precision and vice versa. We do so by three application examples and a simulation experiment. In the first two applications, the consideration of the AE uncertainty is largely unimportant for *ΔΔC*_*q*_ inference due to a large number of dilution steps and well-determined AE. In the last application, we see that the AE uncertainties have a drastically different impact on *ΔΔC*_*q*_ inference. In a simulation study, we show that the methods proposed indeed control the false positive rate better than the conventional approach and provide further insight into the problem.

In the first application, we also verify that multiple myeloma cancer cell lines differentially express the *MGST1* gene depending on the abundance of culture initiating cells. In the second application, the approaches are also used to design and analyze a study which results turned out to support the hypothesis of [22] that miRNA-127 is differentially expressed between testicular and nodal DLBCL.

## Methods

### Observational model

In order to approximate the standard error of the AE adjusted *ΔΔC*_*q*_ we model the amplification process in the following way

(1) $$ F_{C_{q}} = \kappa N_{0} (2^{\alpha})^{C_{q}},   $$

where $F_{C_{q}}$ is the fluorescence measurement at the *C*_*q*_’th cycle, *κ* is a sample-specific proportionality constant, *N*_0_ is the number of transcripts of interest in the initial sample before amplification, and 2^*α*^ is the AE from which *α* is interpreted as the percentage growth on the log scale. In practice, the transcript abundance level is determined by the cycle *C*_*q*_ for which a given fluorescence measurement $F_{C_{q}}$ is reached. Please note, by sample-specific we mean inter sample variations, like pipetting error, which should be accounted for by the reference genes. We rearrange (1) and notice that *C*_*q*_ can be expressed as $\alpha C_{q} = \log _{2} F_{C_{q}} - \log _{2} \kappa N_{0}$. In order to estimate the relative amount of target (tgt) gene transcripts between case and control (ctrl) samples, we assume the amount of the reference (ref) gene template is the same in both the case and the control, *N*_0,ref,case_=*N*_0,ref,ctrl_, and that the AE only vary between the target and reference gene. We then arrive at the following expression for the $\log _{2}$-fold change of the target gene template between case and controls:

$${} {\fontsize{9pt}{9pt}{\begin{aligned} \log_{2}\!\left(\frac{N_{0,\text{tgt},\text{case}}}{N_{0,\text{tgt},\text{ctrl}}}\!\right) &= \log_{2} \kappa_{\text{case}} N_{0,\text{tgt},\text{case}} -\log_{2} \kappa_{\text{case}} N_{0,\text{ref},\text{case}} \\ &\quad - \log_{2} \kappa_{\text{ctrl}} N_{0,\text{tgt},\text{ctrl}} + \log_{2} \kappa_{\text{ctrl}} N_{0,\text{ref},\text{ctrl}} \\ &= - \left\{{\vphantom{(\alpha_{\text{tgt}} C_{q,\text{tgt},\text{ctrl}} - \alpha_{\text{ref}} C_{q,\text{ref},\text{ctrl}})}}\left(\alpha_{\text{tgt}} C_{q,\text{tgt},\text{case}} - \alpha_{\text{ref}} C_{q,\text{ref},\text{case}}\right)\right. \\ &\quad\left.- \left(\alpha_{\text{tgt}} C_{q,\text{tgt},\text{ctrl}} - \alpha_{\text{ref}} C_{q,\text{ref},\text{ctrl}}\right)\right\}, \end{aligned}}} $$

assuming that the *C*_*q*_-values have been determined by a common florescence level $F_{C_{q}}$. This method of estimating the log relative abundance between case and controls is often referred to as the *ΔΔC*_*q*_-method [23], after the double difference appearing in the expression:

(2) $$ \begin{aligned} \Delta\Delta C_{q} &:= \left(\alpha_{\text{tgt}}C_{q,\text{tgt},\text{case}} {-} \alpha_{\text{ref}}C_{q,\text{ref},\text{case}}\right)\\ &\quad{-} \left(\alpha_{\text{tgt}}C_{q,\text{tgt},\text{ctrl}} {-} \alpha_{\text{ref}}C_{q,\text{ref},\text{ctrl}}\right).  \end{aligned}  $$

Thus we have $2^{-\Delta \Delta C_{q}}\phantom {\dot {i}\!}$ as the relative abundance of the original target transcript corrected for the AE.

### Statistical model

We study the problematic aspects of ignoring the uncertainty of the AE estimate. Note, however, that this problem persists for *all* statistical models and methods which naïvely ‘plug-in’ the AE estimate from dilution curves into formulae concerning the *ΔΔC*_*q*_.

For ease of notation we use the abbreviations *i*∈{tgt,ref} for gene types target and reference; *j*∈{case,ctrl,std} for sample types case, control, and standard curve; $s\in \{1,\dots,n_{ij}\}$ for samples in the *ij*’th group; and $k\in \{0,\dots,K_{ijs}\}$ for dilution steps for each sample. To estimate *ΔΔC*_*q*_ of (2), estimates of *α*_*i*_ are needed. A popular way of estimating the AE is by ordinary linear regression. I.e. by regressing *C*_*q*,*ij*_ against a series of increasing values 0=*x*_1_<⋯<*x*_*K*_, defined by $N_{0,ijk} = N_{0,ij}2^{-x_{k}}\phantom {\dot {i}\!}$, and naïvely plugging $\hat {\alpha }_{i}$ into (2) and thus disregarding its uncertainty. Here, *k* denotes the dilution step and *x*_*k*_ the number of 2-fold dilutions (e.g. *x*_1_=1 means the first dilution step halves the original concentration). The *C*_*q*,*ij*_-values and *α*_*i*_ can then be estimated simultaneously when formulated as a LMM [24];

(3) $$  C_{q,ijsk} = \mu_{ij} + A_{js} + \gamma_{i} x_{k} + \epsilon_{ijsk},  $$

where *μ*_*ij*_ is the group means, *A*_*js*_ is a random effect from sample *s* under the *j*’th sample type, and $\gamma _{i} = \alpha _{i}^{-1}$ is the inverse $\log _{2}$-AE. That is,

$$ \text{AE}_{i} = 2^{\frac{1}{\gamma_{i}}} = 2^{\alpha_{i}}. $$

The random effects *A*_*js*_ of (3) are ${\cal {N}}\left (0,{\sigma _{S}^{2}}\right)$-distributed and the error terms *ε*_*ijsk*_ are independent and ${\cal {N}}\left (0,{\sigma ^{2}_{j}}\right)$-distributed with a sample type specific variance ${\sigma ^{2}_{j}}$. The random effects account for the paired samples across tgt/ref for each *j*. LMMs provide a more correct quantification of the sources of variation and thereby a more correct estimate of the uncertainty of *μ*_*ij*_ and their derived quantities.

In one application we shall relax the assumption that the AE is independent of *j* and consider group-specific AEs $\alpha _{ij} = \gamma _{ij}^{-1}$.

Although, variation due to technical replicates should be modeled in (3) as an additional random effect term, we average out technical replicates for simplicity. For further simplicity of this paper, we refrained from using multiple reference genes simultaneously in the *ΔΔC*_*q*_ estimation although our the framework and methods easily extends to this case.

### Inference for *ΔΔC*_*q*_ by the delta method and Monte Carlo integration

We first consider hypothesis testing and confidence intervals for *ΔΔC*_*q*_ by the statistical delta method. Let the maximum likelihood estimates of the fixed effects

$$ \boldsymbol{\theta} = \left(\mu_{\text{tgt},\text{case}}, \mu_{\text{tgt},\text{ctrl}}, \gamma_{\text{tgt}}, \mu_{\text{ref},\text{case}}, \mu_{\text{ref},\text{ctrl}}, \gamma_{\text{ref}}\right)^{\top} $$

be denoted by $\hat {\boldsymbol {\theta }} = (\hat {\mu }_{\text {tgt},\text {case}}, \hat {\mu }_{\text {tgt},\text {ctrl}}, \hat {\gamma }_{\text {tgt}}, \hat {\mu }_{\text {ref},\text {case}},\hat {\mu }_{\text {ref},\text {ctrl}}, \hat {\gamma }_{\text {ref}})^{\top }$. We wish to test the hypothesis *H*_0_:*c*(***θ***)=0, where *c* is the continuously differentiable function of the fixed effects given by

(4) $$ \begin{aligned}  c(\boldsymbol{\theta}) &= \left\{ \left(\mu_{\text{tgt},\text{case}} \gamma_{\text{tgt}}^{-1} - \mu_{\text{ref},\text{case}} \gamma_{\text{ref}}^{-1}\right)\right.\\ &\quad\left.- \left(\mu_{\text{tgt},\text{ctrl}} \gamma_{\text{tgt}}^{-1} - \mu_{\text{ref},\text{ctrl}} \gamma_{\text{ref}}^{-1}\right)\right\}. \end{aligned}  $$

The main task of this paper is to approximate the standard error of $c(\hat {\boldsymbol {\theta }})$ and thereby account for the uncertainty of *ΔΔC*_*q*_. That is, the standard error,

(5) $$  \text{se}(\hat{\boldsymbol{\theta}}) = \sqrt{\mathbb{V}\text{ar}\big[c(\hat{\boldsymbol{\theta}})\big]},  $$

is of central interest. The standard error is used in the statistic for testing *H*_0_ given by $t = c(\hat {\boldsymbol {\theta }})/\text {se}(\hat {\boldsymbol {\theta }})$. From a first order Taylor expansion of *c* around ***θ***,

$$ c(\hat{\boldsymbol{\theta}}) \approx c(\boldsymbol{\theta}) + \nabla c(\boldsymbol{\theta})^{\top} (\hat{\boldsymbol{\theta}} - \boldsymbol{\theta}), $$

where $\mathbb {V}\text {ar}[\!\hat {\boldsymbol {\theta }}]$ is the variance-covariance matrix and ∇*c*(***θ***) is the gradient vector, the variance can be obtained by

$$ \mathbb{V}\text{ar}\big[c(\hat{\boldsymbol{\theta}})\big] \approx \nabla c(\boldsymbol{\theta})^{\top} \mathbb{V}\text{ar}[\!\hat{\boldsymbol{\theta}}] \nabla c(\boldsymbol{\theta}). $$

Notice, this expression coincides with the formula for error propagation in Tellinghuisen and Speiss ([10], p. 95). Hence we approximate *t* by

(6) $$ t = \frac{c(\hat{\boldsymbol{\theta}})}{\sqrt{\nabla c(\hat{\boldsymbol{\theta}})^{\top} \mathbb{V}\text{ar}[\!\hat{\boldsymbol{\theta}}] \nabla c(\hat{\boldsymbol{\theta}})}}.   $$

According to ([24], Section 2.4.2), *t* is approximately *t*-distributed with *η* degrees of freedom. The degrees of freedom of multilevel mixed effects models are non-trivial to obtain in general. We do not pursue this further and restrict ourselves to the case of balanced experimental designs where *η* is obtained relatively straight-forwardly.

On the basis of (6), an approximate (1−*α*)100 *%* confidence interval of *c*(***θ***) can then be given by

$$ c(\hat{\boldsymbol{\theta}}) \pm t_{\alpha/2,\eta}\sqrt{\nabla c(\hat{\boldsymbol{\theta}})^{\top}\mathbb{V}\text{ar}[\!\hat{\boldsymbol{\theta}}]\nabla c(\hat{\boldsymbol{\theta}})}. $$

Likewise, *p*-values can be obtained by computing $P(\lvert t \rvert > T)$ where *T* is *t*-distributed with *η* degrees of freedom.

Alternatively to (6), the variance $\mathbb {V}\text {ar}\big [c(\hat {\boldsymbol {\theta }})\big ]$ can be evaluated by Monte Carlo integration. One way is to simulate a large number *N* of parameters ***θ***_1_,…,***θ***_*N*_ from a multivariate normal distribution using the estimated parameters ${\cal {N}}_{6}({\hat {\boldsymbol {\theta }}}, {\mathbb {V}\text {ar}}[\!{\hat {\boldsymbol {\theta }}}])$ and compute the sample variance of *c*(***θ***_1_),…,*c*(***θ***_*N*_).

Both maximum likelihood (ML) and restricted maximum likelihood estimation (REML) of LMMs is implemented in the R-packages lme4 and nlme [24, 25]. The packages readily provides the estimate ${\hat {\boldsymbol {\theta }}}$ and ${\mathbb {V}\text {ar}}[\!{\hat {\boldsymbol {\theta }}}]$ and we use these in the construction of test and confidence intervals for the *ΔΔC*_*q*_ as described above. The needed gradient in (6) is computed straight-forwardly from (4).

We note that the division by *γ*_*j*_ in (4) is problematic as $\hat {\gamma }_{j}$ is normally distributed and values near zero can increase the variance dramatic. In practice, this is only problematic if the standard error of $\hat {\gamma }_{j}$ is sufficiently large. One way to solve this problem is to use the $\log _{2}$ concentration as the response and the *C*_*q*_-values as the explanatory variables in a regression model of the standard curve to estimate *α*_*j*_ directly. This approach is not without conceptual problems as this puts the errors on the explanatory variables. To this end, note that the hypothesis $H_{0}: \gamma _{\text {tgt}}\gamma _{\text {ref}} c(\hat {\boldsymbol {\theta }}) = 0$, can be equivalently tested for which the standard error of the test-statistic can be worked out exactly.

If $\gamma _{\text {tgt}}^{-1}$ and $\gamma _{\text {ref}}^{-1}$ are assumed to be one (or otherwise known) then (4) becomes a simple linear hypothesis for which the standard error is easily calculated. This corresponds to leaving out the terms in (3) involving these parameters and thus ignoring dilution data. If $\gamma _{{\text {tgt}}}^{-1} = \gamma _{{\text {ref}}}^{-1} = 1$ is assumed, we shall refer to the obtained estimate as the naïve LMM. If $\gamma _{{\text {tgt}}}^{-1}$ and $\gamma _{{\text {ref}}}^{-1}$ are assumed known (i.e. disregarding the standard error hereof) we refer to the obtained estimate as the efficiency corrected (EC) estimate. The estimate where the uncertainty of the AE is considered is referred to as efficiency corrected and variance adjusted by either the delta method (EC&VA1) or Monte Carlo integration (EC&VA2).

### Inference for *ΔΔC*_*q*_ by the bootstrap method

We now consider hypothesis testing and confidence intervals for *ΔΔC*_*q*_ by bootstrapping as an alternative approach. The bootstrap, which avoids calculating gradients, is often cited to perform better in small sample situations [26].

The basic idea of the bootstrap is that inference on *ΔΔC*_*q*_ can be conducted by re-sampling the sample data with replacement to obtain an approximate sampling distribution of the statistic and thereby its properties. In the usual qPCR setup with paired samples and dilution data, straight-forward bootstrapping will quickly fail. We propose non-parametric block bootstrap samples for the case-control data generated by sampling matched pairs of tgt/ref genes with replacement for cases and controls, respectively. However, as we have only got a single observation for each dilution step we chose to re-sample residuals from a simple linear regression model and subsequently adding the residuals to the fitted values from the linear regression. Hence the *B* bootstrapped datasets consists of the re-sampled matched pairs and the residual bootstrapped standard curve. For each dataset, $\hat {\delta }_{1} = {\Delta \Delta C_{q}}^{(1)}, \ldots, \hat {\delta }_{B} = {\Delta \Delta C_{q}}^{(B)}$ are computed to obtain the bootstrap distribution from which confidence intervals and *p*-values can be obtained. The standard error of *ΔΔC*_*q*_ is estimated by the sample standard deviation of the bootstrap distribution. A (1−*α*)100 *%* confidence interval can be computed as $ (\hat {\delta }_{(\alpha /2)}, \hat {\delta }_{(1-\alpha /2)}) $ where e.g. $\hat {\delta }_{(\alpha /2)}$ denotes the *α*/2-percentile of $\hat {\delta }_{1}, \ldots, \hat {\delta }_{B}$. The *p*-value for the null hypothesis of *δ*=0 is computed by 


While the bootstrap is an intuitive and excellent method for estimating the standard error, it quickly becomes computationally heavy. The rather complicated designs of qPCR experiments with paired samples, dilution data, and other random effects also makes the bootstrap less attractive as good bootstrap sampling schemes are hard to produce.

Alternatively, parametric bootstrap can be used by simulating datasets from the fitted model. Here, both new random effects and noise terms are realized and added to the fitted values to generate new datasets.

Re-sampling methods for qPCR data have previously been proposed by [27] to infer the relative expression directly by permutation testing. Unlike the permutation testing of [27], the bootstrap is here used to estimate the mean and standard error of *ΔΔC*_*q*_ and not *directly* test the stated hypothesis. The bootstrap approach suggested here also allows for constructing confidence intervals.

## Applications

We applied the described approaches to two qPCR validation experiments regarding culture initiating cells (CICs) in multiple myeloma (MM) and non-coding microRNAs in diffuse large B-cell lymphoma (DLBCL). In both experiments, the *C*_*q*_-values were extracted for both the reference and target transcripts with automatic baseline and threshold selection [28]. We also illustrate the method on a public available qPCR dataset concerning the differential gene expression in arabidopsis thaliana grown under different conditions. In order to gauge the performance of the methods we subsequently performed a simulation study.

### CIC study

#### Introduction

A cell is culture initiating if it can initiate a sustained production of cells when cultured in vitro. The viability potential of a cell population can be assessed by measuring the number of culture initiating cells (CICs). This number can be estimated by a dilution experiment where cells are seeded in decreasing numbers. The ratio of CICs can then be estimated by e.g. Poisson regression [29]. CICs are of particular interest in cancer research as cancers with high culture initiating potential seemingly have stem cell like properties making them resistant towards chemotherapy [30].

In search for genes associated with a high culture initiating potential in MM we made limiting dilution experiments of 14 MM cell lines and divided them into 7 cell lines with low and 7 cell lines with high culture initiating potential. Gene expression profiling by microarrays identified genes *MGST1* and *MMSET* to be differentially expressed between cell lines with high and low abundance of CICs. As gene expression detection by microarrays can be hampered by high false positive rates, the purpose of this experiment was to validate the findings of the association of *MGST1* and *MMSET* with culture initiating potential by qPCR.

#### Sample and data preparation

For this, 8 MM cell lines (AMO-1, KMM-1, KMS-11, KMS-12-PE, KMS-12-BM, MOLP-8, L-363, RPMI-8226) with >10 *%* CICs, and 8 MM cell lines (ANBL-1, KAS-6-1, LP-1, MOLP-2, NCI-H929, OPM-2, SK-MM-2, U-266) with <1 *%* CICs were used. The fraction of CICs was determined by the limiting dilution method, see [29]. Total RNA was isolated from frozen cell culture pellets, using a combined method of Trizol (Invitrogen) and Mirvana spin columns (Ambion). Isolated RNA was reversed transcribed into complementary DNA (cDNA) synthesis using SuperScript III First-Strand Synthesis Supermix (Invitrogen). As input into the total cDNA synthesis of 250 ng total RNA was used. Equal amounts of random hexamers and oligo(dT) were used as primers. Quantitative real-time reverse transcriptase polymerase chain reaction was performed on a Mx3000p qPCR system (Agilent Technologies/Stratgene) using the TaqMan UniversalPCR Master Mix, No AmpErase UNG, and TaqMan gene expression Assays (Applied Biosystems). The following TaqMan Gene Expression Assays were used (Assay ID numbers in parentheses): *MGST1* (Hs00220393_m1), *MMSET* (Hs00983716_m1). The two reference genes beta-actin (*ACTB*) and *GAPDH* were used as endogenous controls, assay IDs 4333762-0912030 and 4333764-1111036, respectively. For each target and reference transcripts a standard curve based on seven 2-fold dilutions was constructed on a reference sample consisting of material from the AMO-1 cell line.

### DLBCL study

#### Introduction

The association between oncogenesis and micro RNAs (miRNAs), short non-coding RNA transcripts with regulatory capabilities, has recently prompted an immense research activity. The possibility to change treatment strategies by transfecting antisense oligonucleotide to control abnormally up-regulated miRNAs in malignant tissue is of particular interest [31]. In that respect up-regulated *miR-127* and *miR-143* in testicular DLBCL have shown treatment changing potential [22]. However, as the number of screened miRNAs was high and the sample size was low in Robertus et al.’s work invoking high risk of false discoveries we set out to validate the differential expression of *miR-127* and *miR-143* in tissues from our own laboratory using our improved qPCR analysis workflow.

#### Sample and data preparation

For this study, DLBCL samples were collected from 8 testicular (case) and 8 nodal (control) paraffin embedded lymphomas at Aalborg University Hospital. The lymphoma tissues were collected during the diagnostic procedure in accordance with the research protocol accepted by the Health Research Ethics Committee for North Denmark Region (Approval N-20100059). Total RNA was isolated using a combined method of Trizol (Invitrogen) and Mirvana spin columns (Ambion). An amount of 10 ng total RNA was synthesized into first strand cDNA in a 15 *μ*L reaction using TaqMan MicroRNA Reverse Transcription Kit (Applied Biosystems) according to the manufactures instruction. In total 1.33 *μ*L cDNA was used as template in the real time PCR amplification performed by Mx3000p QPCR system (Agilent Technologies/Stratgene) with sequence specific TaqMan primers (Applied Biosystems). As reference transcripts we chose *RNU-6B* and *RNU-24*, which were less variable and equally expressed across nodal and extra-nodal samples among a larger list of candidate reference genes. For each target and reference transcripts a standard curve based on seven 2-fold dilutions was constructed on a reference sample consisting of pooled material from all 16 lymphomas.

### Arabidopsis thaliana study

#### Introduction

In order to illustrate the effect of applying variance approximations in a dataset with a limited number of dilution steps and samples we considered the arabidopsis thaliana dataset published by [7]. The dataset contains one gene of interest, *MT7*, potentially differentially expressed under two growth conditions of the plant arabidopsis thaliana and two reference genes ubiquitin (*UBQ*) and tublin.

#### Sample and data preparation

The arabidopsis thaliana plant growth, RNA extraction, and qPCR experiments were carried out as described in [32]. The cDNA was diluted into 1-to-4 and 1-to-16 serial dilutions. Real-time PCR experiments was performed in duplicates for each concentration [7].

Due to the study design, we naturally fitted estimation efficiencies $\gamma _{ij} = \alpha _{ij}^{-1}$ for each group. Because of the few samples we omitted the, in this case, meaningless random sample effect of the LMM.

### Simulation study

In order to properly benchmark statistical test procedures one needs to have an idea of the false positive rate (FPR), or type I error rate, as well as the true positive rate (TPR), or sensitivity. As ground truth is usually not available in non-synthetic data, we use simulation experiments to determine the error rates of the discussed statistical procedures.

In our setting, the FPR of a statistical test is the probability that the test incorrectly will declare a result statistically significant given a vanishing effect size or difference of *c*(*θ*)=0 between case and controls; i.e. $\text {FPR} = P\left (\vert t \vert > t_{1 - \alpha /2,\eta } \vert c(\theta) = 0\right)$. On the other hand the TPR of the statistical test is the probability that the test will correctly declare a result statistically significant given an non-zero effect size *δ*=*c*(*θ*) between case and controls; i.e.$ \text {TPR} = P\left (\vert t \vert > t_{1 - \alpha /2,\eta } \vert c(\theta) = \delta \right)$.

A straightforward way to obtain an estimate of the TPR is to simulate a large number *n* of datasets under the alternative hypothesis of *c*(*θ*)=*δ*, fit the model for each dataset, and compute *t*-values $t_{1}, \dots, t_{n}$. From these *t*-scores the TPR can estimated by 


where  is the indicator function. Hence, the estimated TPR is the fraction of tests correctly declared significant.

Likewise, an estimate of the FPR is obtained by simulating *n* datasets under the null hypothesis of *c*(*θ*)=0 and obtaining *t*-values $t_{1}, \dots,t_{n}$ from which FPR is estimated by 


i.e. the fraction of tests incorrectly declared significant.

Simulating under the log-linear statistical model described above, we estimate the FPR and the TPR for each discussed method under different choices of sample sizes and number of dilutions whilst fixing the size of the sample and experimental variations. These constant sample and experimental variations corresponds to homoscedastic errors on the log-scale. No technical replications are simulated.

## Results

### CIC study

**Fig. 1:**
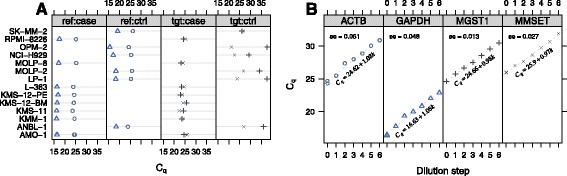
Overview of CIC experiment data. **a** Raw *C*_*q*_-values for different cell lines (samples) for each gene type and sample type. The point type and colour differentiates the different gene types. **b** Dilution data for reference genes (*ACTB*, *GAPDH*) and target genes (*MGST1*, *MMSET*)

The *C*_*q*_-values and dilution curves for the CIC study are depicted in Fig. 1 panels a–b, respectively. The simple linear regressions show well-determined standard curves with small standard errors on the estimate of the slopes.

The values of the considered estimators for *ΔΔC*_*q*_ are seen in Table 1. The table also shows results of tests for difference in gene expression assessed by the *ΔΔC*_*q*_ for both target genes *MGST1* and *MMSET* normalized to each of the reference genes *GAPDH* and *ACTB*. We used four different methods to estimate and perform inference: (1) EC: Efficiency corrected LMM estimate ignoring the uncertainty of the efficiency estimates. (2) EC&VA1: EC and variance adjusted LMM estimate using a first order approximation. (3) EC&VA2: EC and variance adjusted LMM estimate using Monte Carlo integration. (4) Bootstrap: Estimate by the bootstrap described in Section “Inference for *ΔΔC*_*q*_ by the bootstrap method” fitting the LMM and using the EC estimate.

**Table 1:** CIC data: Method comparison for estimating the *Δ* *Δ* *C*_*q*_-value

	Estimate	se	*t*-value	df	*p*-value	LCL	UCL
MGST1 vs GAPDH							
EC	–8.62	1.62	–5.31	21	2.92·10^−5^	–12	–5.24
EC&VA1	–8.62	1.66	–5.18	21	3.89·10^−5^	–12.1	–5.16
EC&VA2	–8.62	1.67	–5.17	21	4.04·10^−5^	–12.1	–5.15
Bootstrap	–8.66	2.06			1.00·10^−3^	–12.5	–4.41
MGST1 vs ACTB							
EC	–8.98	1.61	–5.57	21	1.57·10^−5^	–12.3	–5.63
EC&VA1	–8.98	1.65	–5.45	21	2.08·10^−5^	–12.4	–5.56
EC&VA2	–8.98	1.65	–5.45	21	2.10·10^−5^	–12.4	–5.55
Bootstrap	–8.98	2.09			1.00·10^−3^	–12.7	–4.48
MMSET vs GAPDH							
EC	0.679	0.585	1.16	21	2.59·10^−1^	–0.538	1.9
EC&VA1	0.679	0.587	1.16	21	2.60·10^−1^	–0.541	1.9
EC&VA2	0.679	0.589	1.15	21	2.62·10^−1^	–0.545	1.9
Bootstrap	0.688	0.678			3.12·10^−1^	–0.656	2
MMSET vs ACTB							
EC	0.318	0.962	0.331	21	7.44·10^−1^	–1.68	2.32
EC&VA1	0.318	0.962	0.331	21	7.44·10^−1^	–1.68	2.32
EC&VA2	0.318	0.964	0.33	21	7.45·10^−1^	–1.69	2.32
Bootstrap	0.342	0.987			7.05·10^−1^	–1.68	2.13

*EC* efficiency corrected LMM estimate ignoring the uncertainty of the efficiency estimates. *EC&VA1* EC and variance adjusted LMM estimate using the delta method. *EC&VA2* EC and variance adjusted LMM estimate using Monte Carlo integration. *Bootstrap* estimate by the bootstrap described in Section “Inference for *ΔΔC*_*q*_ by the bootstrap method” fitting the LMM and using the EC estimate. Bootstrap shows the mean and standard deviation of 2000 bootstrap samples using the EC estimate. The last two columns show the 95 *%* lower and upper confidence interval limits

Consider the first section of Table 1 where tgt *MGST1* is normalized against the reference *GAPDH*. The tests for a vanishing *ΔΔC*_*q*_ are all highly significant with comparable 95 *%* CIs. As expected, the efficiency corrected estimates are unchanged due to the variance adjustment, and only the standard deviation of the estimate is increased. The increase of the standard error is very small resulting in small but unimportant increases of the absolute *t*- and *p*-values. The results remain significant for the *MGST1* gene. Very similar results are obtained if *ACTB* is used as reference. In conclusion, there is good evidence that *MGST1* is differentially expressed between cell lines with high and low abundance of CICs.

For the target gene *MMSET* normalized with respect to both reference genes, all estimates are not significantly different from zero. Again, the various methods all agree and no substantial inter-method differences are seen and we find no evidence for differential expression of *MMSET* between cell lines with high and low abundance of CICs.

In all instances, the bootstrap distribution mean agree well with the estimates obtained using the delta or Monte Carlo methods while it seems to provide a larger standard error. This tendency have one or more probable explanations. The first order delta method and Monte Carlo approximations may underestimate the standard error and the bootstrap, corresponding to a higher order method, more correctly quantify it. More likely, the data deviate slightly from the model assumptions and the bootstrap is sensitive to this slight misspecification.

We see that the large number of dilution steps, as recommended and expected, ensures a low impact of the AE on the standard error and thus on the inference of the *ΔΔC*_*q*_.

### DLBCL study

**Fig. 2:**
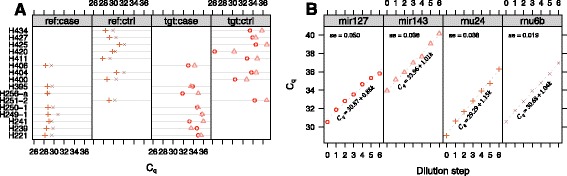
Overview of DLBCL testis data. **a** Raw *C*_*q*_-values for different patient samples for each gene type and sample type. The point type and colour differentiates the different gene types. **b** Dilution data for reference genes (*RNU-24*, *RNU-6B*) and target genes (*miR-127*, *miR-143*)

The *C*_*q*_-values and dilution curves for the DLBCL study are depicted in Fig. 2, panels a–b, respectively. Analogous to the previous section, the differences in gene expressions assessed by the *ΔΔC*_*q*_ for the target genes *miR-127* and *miR-143* with respect to each reference gene rnu6b and rnu24 were estimated using the four different methods. Again 2000 bootstrapped samples were used. The results are seen in Table 2.

**Table 2:** DLBCL data: Method comparison for estimating the *Δ* *Δ* *C*_*q*_-value

	Estimate	se	*t*-value	df	*p*-value	LCL	UCL
mir127 vs rnu6b							
EC	2.67	1.13	2.37	22	2.68·10^−2^	0.336	5.01
EC&VA1	2.67	1.13	2.37	22	2.71·10^−2^	0.331	5.01
EC&VA2	2.67	1.13	2.36	22	2.75·10^−2^	0.325	5.02
Bootstrap	2.68	1.05			1.00·10^−3^	0.876	4.82
mir127 vs rnu24							
EC	2.38	1.08	2.2	22	3.87·10^−2^	0.136	4.63
EC&VA1	2.38	1.09	2.19	22	3.91·10^−2^	0.13	4.64
EC&VA2	2.38	1.09	2.19	22	3.94·10^−2^	0.126	4.64
Bootstrap	2.42	1.18			1.00·10^−2^	0.416	5.02
mir143 vs rnu6b							
EC	1.17	0.846	1.38	22	1.82·10^−1^	-0.589	2.92
EC&VA1	1.17	0.846	1.38	22	1.82·10^−1^	-0.59	2.92
EC&VA2	1.17	0.847	1.37	22	1.83·10^−1^	-0.592	2.92
Bootstrap	1.15	0.794			1.44·10^−1^	-0.341	2.7
mir143 vs rnu24							
EC	0.878	0.81	1.08	22	2.90·10^−1^	-0.801	2.56
EC&VA1	0.878	0.81	1.08	22	2.90·10^−1^	-0.802	2.56
EC&VA2	0.878	0.811	1.08	22	2.90·10^−1^	-0.803	2.56
Bootstrap	0.897	0.822			2.67·10^−1^	-0.603	2.58

*EC* efficiency corrected LMM estimate ignoring the uncertainty of the efficiency estimates. *EC&VA1* EC and variance adjusted LMM estimate using the delta method. *EC&VA2* EC and variance adjusted LMM estimate using Monte Carlo integration. *Bootstrap* Estimate by the bootstrap described in Section “Inference for *ΔΔC*_*q*_ by the bootstrap method” fitting the LMM and using the EC estimate. Bootstrap shows the mean and standard deviation of 4 bootstrap samples using the EC estimate. The last two columns show the 95 *%* lower and upper confidence interval limits

We notice the efficiency corrected estimates are exactly equal with and without variance adjustment, while the standard deviation of the estimate and the *p*-values are higher for the adjusted values as expected. The size of the increase is again undramatic hinting at well determined AE using the dilution curves.

For all combinations of reference genes the estimates for *miR-127* are significantly different from zero at the usual 5 *%* significant level, but not at the 1 *%* significance level. The *miR-143* estimates are not significantly different from zero. Despite the very small increase in standard error, the *p*-values increase at the second digit.

The bootstrap method provides a standard deviation similar to the delta method and Monte Carlo integration for both *miR-127* and *miR-143*.

Regarding the biological interest, we conclude there is evidence for a difference in *miR-127* expression between testicular and nodal DLBCL whilst the data is not compatible with difference in *miR-143* expression. While the AE estimate had no influence in these cases a change in significance is easily imagined in other cases.

### Arabidopsis thaliana data

**Fig. 3:**
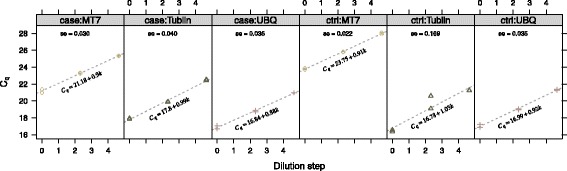
Overview of Arabidopsis thaliana data [7]. *C*_*q*_-values against the dilution step for case and control samples. Dilution data are present for both the target (*MT7*) and reference genes (Tublin, *UBQ*)

The *C*_*q*_-values and dilution data for the arabidopsis thaliana data are shown in Fig. 3.

The estimated difference in gene expression between case and control of the target gene *MT7* normalized to either reference (Tublin or *UBQ*) is seen in Table 3. The table shows the efficiency corrected method with and without variance adjustment by the delta method. In both cases, we see a dramatic increase in the standard error, *p*-values, and size of the confidence intervals. When using variance adjustment there is no longer a highly statistical significant difference in *MT7* expression between case and ctrl growth conditions.

**Table 3:** Arabidopsis thaliana data [7]: Method comparison for estimating the *Δ* *Δ* *C*_*q*_-value

	Estimate	se	*t*-value	df	*p*-value	LCL	UCL
MT7 vs Tublin							
EC	-4.374	0.4319	-10.13	4	5.353·10^−4^	-5.573	-3.174
EC&VA1	-4.374	3.788	-1.155	4	3.126·10^−1^	-14.89	6.144
MT7 vs UBQ							
EC	-3.381	0.137	-24.67	4	1.601·10^−5^	-3.761	-3
EC&VA1	-3.381	1.351	-2.503	4	6.658·10^−2^	-7.132	0.3699

*EC* efficiency corrected LMM estimate ignoring the uncertainty of the efficiency estimates. *EC&VA1* EC and variance adjusted LMM estimate using the delta method. The last two columns show the 952000 lower and upper confidence interval limits

The results may be surprising at first sight when considering the relatively small standard errors of the slopes in the simple linear regressions shown in Fig. 3. One might imagine that the uncertainty of the AE is negligible and thus perform the usual analysis. However, we see the contrary for several reasons. First, using only 3 dilutions steps leaves very few degrees of freedom left in each group as we are left with few samples and a high number of parameters to be estimated. Secondly, as dilution curves are used for each group the four group-specific AE estimates will all contribute to increasing the standard error of the *ΔΔC*_*q*_. While this example was selected as a worst-case scenario, it should illustrate that although the standard curves are seemingly well determined, it is hard to intuitively predetermine the combined effect on the standard error of *ΔΔC*_*q*_.

We note here, that no pre-averaging of the technical replicates for each concentration was done. Instead, the technical replicates where modeled as a random effect.

### Simulation study

First, we present results of a simulation study for a two-sided test for the null hypothesis of a vanishing *ΔΔC*_*q*_ at a 5 *%* significance level. We simulated 2000 datasets under both the null and alternative hypothesis with 6 samples in each case and control group and standard curves with 6 dilution steps. The effect size under the alternative was set to *δ*=10/9. The sample and experimental standard deviations were set to *σ*_*S*_=1 and *σ*=1, respectively. The AE for the target and reference genes were set to 0.80 and 0.95, respectively. The parameters were primarily chosen to conveniently yield estimates and error rates on a sensible scale whilst secondarily being comparable to estimated quantities in the applications.

The four discussed methods were applied to the 2×2000 datasets and the *p*-value testing the null hypothesis were computed. The results of these tests are summarized in Table 4 from which the FPR and TPR can be computed at the 5 *%* cutoff. From Table 4, we see the estimated FPRs are 0.073, 0.053, and 0.083 for the efficiency corrected LMM (EC), the efficiency corrected LMM with variance adjustment using the delta method (EC&VA1), and the bootstrap, respectively. We omitted EC&VA by Monte Carlo integration here due to the computational cost and the similar results with EC&VA1 in the previous. As expected, the EC method does not control the FPR at the 5 *%*-level. The variance adjusted estimator is consistent with controlling the FPR at the 5 *%* level. By construction, the variance adjusted will always perform at least as good as the EC in terms of FPR. Surprisingly, the bootstrap has the worst performance in terms of FPR.

**Table 4:** Contingency tables for the different estimators for at 5 *%* *p*-value threshold

	EC			EC&VA1			Bootstr.	
	*H*_0_	*H*_*A*_		*H*_0_	*H*_*A*_		*H*_0_	*H*_*A*_
*p*-values								
*p*≥0.05	1854	1235		1894	1365		1834	1268
*p*<0.05	146	765		106	635		166	732

The used estimators are the LMM with efficiency correction (EC), the LMM with EC and variance adjustment (EC&VA), and the bootstrapped LMM approach

Secondly, the TPR are estimated to be 0.3825, 0.3175, 0.366 for three methods EC, EC&VA1, Bootstr., respectively. As expected, we notice that an improved FPR comes a the cost of a decreased TPR for a given statistical procedure.

**Fig. 4:**
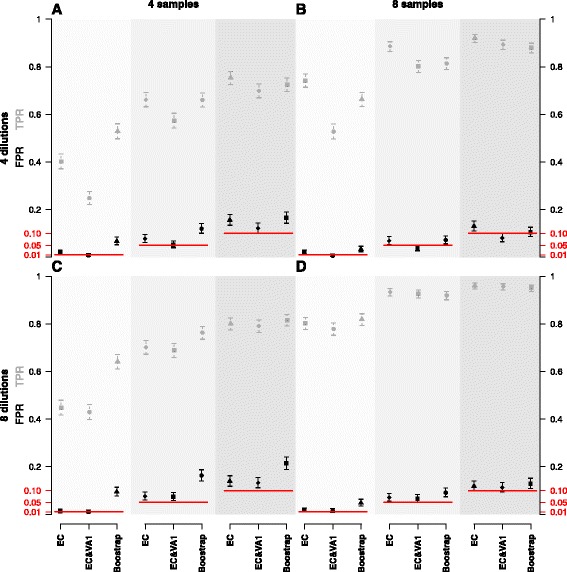
Method performance. Plot of the false positive rates (FPR, *black*) and true positive rates (TPR, *grey*) and their 95 *%* confidence intervals achieved simulation experiments for each method at various *p*-value cut-offs (0.05, 0.01, 0.1) shown by solid red horizontal lines. The FPR and TPR are computed completely analogous to Table 4. The rates are plotted for each combination of 4 or 8 samples with 4 or 8 fold dilution curves

The above simulations were repeated for sample sizes 4 or 8 in both case and control groups in combination with 4 or 8 dilution steps with the same simulation parameters. Figure 4 shows the performance of the methods in terms of FPR and TPR. Each panel corresponds to a given number of samples and dilutions. In each panel the *p*-value cut-off is varied between 0.01, 0.05, and 0.1. Overall, we see that the EC&VA estimate is the only procedure consistent with controlling the FPR at the nominal chosen significance level. Likewise, for many dilutions, the difference between the EC and EC&VA procedures diminish as the uncertainty of the AE is relatively low. As expected a decrease in FPR corresponds to a decrease in TPR.

**Fig. 5:**
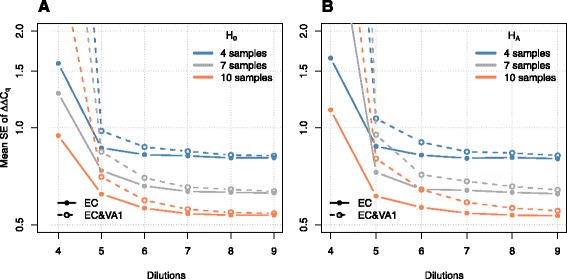
Standard error comparison. The mean standard error of the *Δ* *Δ* *C*_*q*_ for two methods (EC and EC&VA1) over 2000 repeated simulations under the null (panel **a**) and alternative hypothesis (panel **b**) as a function of the number of dilution steps for a different number of samples in each group

To gauge when the standard error of (5) is determined with adequate precision, we simulated 2×2000 datasets and computed the mean standard error of the *ΔΔC*_*q*_ for the EC and EC&VA procedures as a function of the number of dilutions and samples. We varied the number of dilutions in the range 4–9 for a number of samples in the range 4–10 with the same settings as above. Figure 5 shows these results. As expected, increasing the number of samples or the number of dilutions yield a smaller standard error. Also unsurprising and as already seen in the applications, the differences in the standard error for the EC and EC&VA methods are very substantial for a small number of dilutions and vanish as the number of dilutions steps increase. The differences in the standard error seems to be larger under the alternative than the null hypothesis. Similar figures might also aid in designing qPCR experiments and help determine if investing in additional dilutions or samples is preferable—obviously with properly chosen simulation parameters in the given context.

## Discussion and conclusion

The commonly used efficiency corrected *ΔΔC*_*q*_ and many other approaches to the analysis of qPCR data disregards the uncertainty of the estimated AE leading to increased false positive rates. As qPCR experiments are often used for validation this is highly undesirable. Our primary approach based on the statistical delta-method to approximate the variance of the efficiency adjusted *ΔΔC*_*q*_, shows that it is possible to perform statistical inference about qPCR experiments whilst more properly accounting for the AE uncertainty. We stress that the problem is not limited to the *ΔΔC*_*q*_ statistic, but all statistics that depend on the AE.

In this paper, we focus on the widely used dilution curve approach as it is complex enough to capture the most important variations but also simple enough to be easily interpretable. This is probably also the reason dilution curves, when carefully used, are still very popular in qPCR experiments. However, this approach has been criticized in the literature as it relies on a number of assumption [12–15]. First, the dilution curve approach assumes the AE to be constant up to the *C*_*q*_’th cycle. A way to check this is to assess whether the dilution curves are reasonably linear, as non-constant behaviour up to the *C*_*q*_’th cycle in our dynamic range would cause the dilution curves to level off. In our data examples, we do not see any violation of the linear behaviour. Secondly, one also assumes the AE varies between wells. However, if we assume that the actual reciprocal AE *γ*_*A*_ varies around a true reciprocal AE value *γ*, by a random variation *Γ*, say, which could be assumed to be Gaussian distributed with mean 0 and variance $\sigma _{\Gamma }^{2}$ one arrives at the following model for *C*_*q*_

$$\begin{array}{*{20}l} C_{q} &= \mu + (\gamma + \Gamma)N_{0} + \epsilon\\ &= \mu + \gamma N_{0} + \underbrace{(\epsilon+\Gamma N_{0})}_{\text{error term}} \end{array} $$

From this follows the proposed cycle dependent variation is captured by the error term in the LMM. We note, however, due to the multiplication with *N*_0_ a variance heterogeneity could be present. We have therefore assessed the LMMs by plotting the residuals against the fitted values and noticed no variance heterogeneity to be present. In conclusion we find our model sufficient to capture the variation for the problems we have at hand.

In practice, the approach was used to: (1) validate that *MGST1* is differentially expressed between MM cell lines of high and low abundance of CICs, (2) analyze and study the hypothesis that miRNA-127 is differentially expressed between testicular and nodal DLBCL, and (3) illustrate the effect of a small number of dilution steps.

In the latter application, we saw a dramatic increase in the standard error of the estimate when the variance approximation was introduced, potentially leading to a change of significance for the presented dataset depending on the desired significance level. This illustrates the importance of considering the AE uncertainty when conducting AE correction of qPCR experiments.

Although not in the context of *ΔΔC*_*q*_ estimation, Tellinghuisen and Speiss [10] concluded that uncertainty as well as bias of the AE estimate substantially impacts subsequent quantities. They highlight that some methods achieve very good performance as measured by the low standard errors by tacitly assuming the AE known and fixed to 2. This is an unsurprising consequence as it is essentially the same as disregarding the AE uncertainty. We also note, as seen in this paper, that a low standard error in itself is not always a proper benchmark of procedures.

Problems with uncertainty in AE estimates should be handled by establishing well-estimated dilution curves as argued elsewhere [6], however even in this case the presented method also allows for design guidelines for power calculations and assessing the influence of the estimated dilution curves.

It is also noteworthy that model based estimation of the *ΔΔC*_*q*_ also allows for model checking by e.g. residual plots.

Lastly, we note that the algorithm [28] we used for threshold selection and *C*_*q*_-value extraction in the CIC and DLBCL studies may not be optimal—cf. [9, 33], and improvements by [34]—as it can be affected by the AE.

Nonetheless, this has no bearing on the stated problem of this paper. The estimated standard error of *ΔΔC*_*q*_ is still affected in a similar manner by the uncertainty of the AE and thus too optimistic.

Despite the extensive use of qPCR, more statistical research is needed to establish qPCR more firmly as a gold standard to reliably quantify abundances of nucleic acids. Researchers analyzing qPCR experiments need to model their experiments in detail, e.g. via linear or non-linear (mixed) models, as the propagation of uncertainty needs to be carefully assessed and accounted for. This is necessary for making valid inferences and upholding the common statistical guarantees often erroneously assumed to be automatically fulfilled. We recommend the conservative and proper approach of *always* accounting for the uncertainty of the AE.

### Supplementary material and software

The statistical analysis were done using the programming language Rv3.2.3 [35] using lme4. All data, R code, LaTeX, and instructions for reproducing this present paper and results are freely available at http://github.org/AEBilgrau/effadj/ using knitr, an extension of Sweave [36, 37]. Functionality from the packages Hmisc [38], lattice (and latticeExtra) [39], epiR [40], snowfall [41], and GMCM [42], were used for tables, figures, FDR/TPR confidence intervals, parallel execution of simulations, and multivariate normal simulations, respectively.

## References

[1] Rabinow P (1996). Making PCR: A Story of Biotechnology.

[2] Pfaffl MW (2001). A new mathematical model for relative quantification in real-time RT-PCR. Nucleic Acids Res.

[3] Rao X, Lai D, Huang X (2013). A new method for quantitative real-time polymerase chain reaction data analysis. J Comput Biol.

[4] Rao X, Huang X, Zhou Z, Lin X (2013). An improvement of the 2^(-delta delta ct) method for quantitative real-time polymerase chain reaction data analysis. Biostatistics, Bioinformatics.

[5] Bustin SA, Benes V, Garson JA, Hellemans J, Huggett J, Kubista M, Mueller R, Nolan T, Pfaffl MW, Shipley GL (2009). The MIQE guidelines: Minimum information for publication of quantitative real-time pcr experiments. Clin Chem.

[6] Bustin SA (2010). Why the need for qPCR publication guidelines?–the case for MIQE. Methods.

[7] Yuan JS, Wang D, Stewart CN (2008). Statistical methods for efficiency adjusted real-time pcr quantification. Biotechnol J.

[8] Nordgård O, Kvaløy JT, Farmen RK, Heikkilä R (2006). Error propagation in relative real-time reverse transcription polymerase chain reaction quantification models: The balance between accuracy and precision. Anal Biochem.

[9] Tellinghuisen J, Spiess AN (2014). Comparing real-time quantitative polymerase chain reaction analysis methods for precision, linearity, and accuracy of estimating amplification efficiency. Anal Biochem.

[10] Tellinghuisen J, Spiess AN (2014). Statistical uncertainty and its propagation in the analysis of quantitative polymerase chain reaction data: Comparison of methods. Anal Biochem.

[11] Tellinghuisen J, Spiess AN (2015). Bias and imprecision in analysis of real-time quantitative polymerase chain reaction data. Anal Chem.

[12] Peccoud J, Jacob C (1996). Theoretical uncertainty of measurements using quantitative polymerase chain reaction. Biophys J.

[13] Jacob C, Peccoud J (1998). Estimation of the parameters of a branching process from migrating binomial observations. Adv Appl Probab.

[14] Lalam N, Jacob C, Jagers P (2004). Modelling the pcr amplification process by a size-dependent branching process and estimation of the efficiency. Adv Appl Probab.

[15] Lalam N, Jacob C (2007). Bayesian estimation for quantification by real-time polymerase chain reaction under a branching process model of the dna molecules amplification process. Math Popul Stud.

[16] Svec D, Tichopad A, Novosadova V, Pfaffl MW, Kubista M (2015). How good is a pcr efficiency estimate: Recommendations for precise and robust qpcr efficiency assessments. Biomolecular Detection Quantification.

[17] Andersen CL, Jensen JL, Ørntoft TF (2004). Normalization of real-time quantitative reverse transcription-pcr data: A model-based variance estimation approach to identify genes suited for normalization, applied to bladder and colon cancer data sets. Cancer Res.

[18] Abruzzo LV, Lee KY, Fuller A, Silverman A, Keating MJ, Medeiros LJ, Coombes KR (2005). Validation of oligonucleotide microarray data using microfluidic low-density arrays: A new statistical method to normalize real-time RT-PCR data. Biotechniques.

[19] Fu WJ, Hu J, Spencer T, Carroll R, Wu G (2006). Statistical models in assessing fold change of gene expression in real-time RT -PCR experiments. Comput Biol Chem.

[20] Steibel JP, Poletto R, Coussens PM, Rosa GJ (2009). A powerful and flexible linear mixed model framework for the analysis of relative quantification RT-PCR data. Genomics.

[21] Matz MV, Wright RM, Scott JG (2013). No control genes required: Bayesian analysis of qRT-PCR data. PloS one.

[22] Robertus JL, Harms G, Blokzijl T, Booman M, de Jong D, van Imhoff G, Rosati S, Schuuring E, Kluin P, van den Berg A (2009). Specific expression of miR-17-5p and miR-127 in testicular and central nervous system diffuse large B-cell lymphoma. Modern Pathol.

[23] Livak KJ, Schmittgen TD (2001). Analysis of relative gene expression data using real-time quantitative PCR and the $2^{-\Delta \Delta C_{q}}\phantom {\dot {i}\!}$ method. Methods.

[24] Pinheiro JC, Bates DM (2000). Mixed-effects Models in S and S-PLUS.

[25] Bates D, Maechler M, Bolker B, Walker S. lme4: Linear Mixed-effects Models Using Eigen and S4. 2014. R package v1.1-7. http://CRAN.R-project.org/package=lme4.

[26] Efron B (1982). The Jackknife, the Bootstrap and Other Resampling Plans. vol. 38. SIAM.

[27] Pfaffl MW, Horgan GW, Dempfle L (2002). Relative expression software tool (restⒸ) for group-wise comparison and statistical analysis of relative expression results in real-time pcr. Nucleic Acids Res.

[28] Mx, 3000P. Mx3000P and Mx3005P QPCR Systems. Setup and User’s Guide: Agilent; 2013.

[29] Lefkovits I, Waldmann H (1999). Limiting Dilution Analysis of Cells of the Immune System.

[30] Chen K, Huang Y-h, Chen J-l (2013). Understanding and targeting cancer stem cells: Therapeutic implications and challenges. Acta Pharmacologica Sinica.

[31] Garzon R, Marcucci G, Croce CM (2010). Targeting micrornas in cancer: rationale, strategies and challenges. Nat Rev Drug Discov.

[32] Yang Y, Yuan J, Ross J, Noel J, Pichersky E, Chen F (2006). An arabidopsis thaliana methyltransferase capable of methylating farnesoic acid. Arch Biochem Biophys.

[33] Ruijter JM, Pfaffl MW, Zhao S, Spiess AN, Boggy G, Blom J, Rutledge RG, Sisti D, Lievens A, De Preter K (2013). Evaluation of qPCR curve analysis methods for reliable biomarker discovery: Bias, resolution, precision, and implications. Methods.

[34] Spiess AN, Deutschmann C, Burdukiewicz M, Himmelreich R, Klat K, Schierack P, Rödiger S (2015). Impact of smoothing on parameter estimation in quantitative dna amplification experiments. Clin Chem.

[35] R Core Team (2012). R: A Language and Environment for Statistical Computing.

[36] Xie Y (2015). Dynamic Documents with R and Knitr. Vol. 29.

[37] Leisch F. Sweave: Dynamic generation of statistical reports using literate data analysis In: Härdle W, Rönz B, editors. Compstat 2002 — Proceedings in Computational Statistics. Physica Verlag, Heidelberg: 2002. p. 575–80. ISBN 3-7908-1517-9.

[38] Harrell JrFE, et al. Hmisc: Harrell Miscellaneous. 2015. R package v3.16-0. http://CRAN.R-project.org/package=Hmisc.

[39] Sarkar D (2008). Lattice: Multivariate Data Visualization with R.

[40] Stevenson M, et al. epiR: Tools for the Analysis of Epidemiological Data. 2015. R package v0.9-62. http://CRAN.R-project.org/package=epiR.

[41] Knaus J. Snowfall: Easier Cluster Computing (based on Snow). 2013. R package v1.84-6. http://CRAN.R-project.org/package=snowfall.

[42] Bilgrau AE, Eriksen PS, Rasmussen JG, Dybkaer K, Johnsen HE, Boegsted M (2016). GMCM: Unsupervised clustering and meta-analysis using gaussian mixture copula models. Journal of Statistical Software.

